# Editorial: Substance abuse and early development

**DOI:** 10.3389/adar.2023.11836

**Published:** 2023-08-09

**Authors:** Anna N. Bukiya, Declan W. Ali

**Affiliations:** ^1^ Department of Pharmacology, Addiction Science and Toxicology, University of Tennessee Health Science Center (UTHSC), Memphis, TN, United States; ^2^ Department of Biological Sciences, University of Alberta, Edmonton, AB, Canada

**Keywords:** prenatal, drug abuse, narcotics, developmental trajectory, developmental toxicity

While misuse of psychoactive substances can be traced back to pre-historic times, their effects on invisible target—such as the developing fetus—only gained recognition within the last century [1–4]. Initial seminal findings in humans and laboratory rodents quickly grew from isolated observations to well-planned, multidisciplinary studies. Despite the involvement of numerous methodologies, large teams of researchers, and widely different experimental organisms, there has been an unusual consensus on the meaning of findings: principally that prenatal exposure to drugs of misuse has deleterious consequences for the developing fetus.

The mechanisms by which prenatal exposure to psychoactive substances alters developmental trajectories are variable, from epigenetic alterations to fine-tuning of blood supply to the brain [5, 6]. In some cases, such as during cannabis exposure, the impact is exacerbated by the vast presence and activity of the endocannabinoid system during early development (Fride, 2008; [7]). In others, such as with alcohol, efforts for elucidating potential mechanisms of alcohol-driven alterations and thus, identifying venues for novel therapeutic options are hindered by the chemical’s simple structure and the abundant sensing sites it can bind to. Additional challenges are presented by the growing trend of substance co-use, such as in cases of simultaneous consumption of alcohol and marijuana [8] or opioid and stimulant products [9]. In these instances, in addition to the effects of individual drugs, their potential interactions must be considered.

The current Special Issue aims at describing recent advances in our understanding of how maternal and paternal substance abuse affects early development of progeny. Contributions into this Special Issue span from methodological advancements, into reviews of current standing in the field, original research report, and novel diagnostic tools. Specifically, Mbolle et al. take the reader on a journey into modern technology for high-resolution imaging of prenatal development. While this task is certainly streamlined in obstetrics clinics, major challenges remain at the bench, as current resolution capabilities are often below the thresholds needed for accurate visualization of fetal structures within small rodent species such as mice which are used for mechanistic studies. Development of deep-tissue high-resolution imaging and accompanying computational reconstruction technology will serve many researchers who are trying to elucidate the underpinnings of prenatal drug exposure effects. Before such methodology could advance the field, a thorough analysis of existing knowledge is needed, so that gaps could be identified and pursued with precision targeting. To address this task, Mulligan and Hamre offer an in-depth review of current standing on the influence of prenatal cannabinoid exposure on early development and beyond. The task is not trivial, as there is a clear difference of opinion between medical professionals and societal attitudes. The authors conclude that while there is evidence that prenatal cannabis exposure might alter developmental trajectories, the cause-effect relationships are not proven and, in most cases, require additional studies.

Strong focus in this Special Issue is maintained on vascular and cerebrovascular consequences of prenatal alcohol exposure. Momin et al. review vascular contributors to the neurobiological effects of prenatal alcohol exposure. This systematic review of the literature leads to a conclusion that although the brain has been traditionally considered as a main target of prenatal alcohol exposure, data from human samples and bench studies document that developing vasculature is equally sensitive to an early alcohol hit. Saha and Mayhan concur with this conclusion and expand upon it by presenting a review of mechanisms that underly cerebral vascular damage by prenatal alcohol exposure and which enable lifelong consequences of this adverse developmental event. Both reviews highlight the fact that our approach to treatment of adverse developmental outcomes should involve a multi-organ strategy.

Waite and Burd conclude the chapter of alcohol prenatal effects with a review of literature on common developmental trajectories and clinical identification of children with fetal alcohol spectrum disorders. They describe current challenges in the diagnosis of fetal alcohol spectrum disorders as opposed to prevalent behavioral disorders. Finally, Fleming et al (*in press*) offer an early, time-efficient screening tool which could assist in efforts to diagnose fetal alcohol spectrum disorders in large cohorts of children. Wide clinical utility of this newly offered tool remains to be documented.

Following current trends of increased opioid use, misuse, and opioid-related deaths, Madurai et al. describe alterations in the peripheral inflammatory and central immune landscapes following prenatal exposure of rats to methadone. The authors conclude that such alterations may underly long-term consequences of developmental brain injury including cognitive and attention deficits. However, identification of altered patterns may also serve as a biomarker and a therapeutic target. Translation of these findings from bench to bedside remains a goal for near future.
